# Serologic Prevalence of Ebola Virus in Equatorial Africa

**DOI:** 10.3201/eid2505.180115

**Published:** 2019-05

**Authors:** Imke Steffen, Kai Lu, Lauren K. Yamamoto, Nicole A. Hoff, Prime Mulembakani, Emile O. Wemakoy, Jean-Jacques Muyembe-Tamfum, Nicaise Ndembi, Catherine A. Brennan, John Hackett, Susan L. Stramer, William M. Switzer, Sentob Saragosti, Guy O. Mbensa, Syria Laperche, Anne W. Rimoin, Graham Simmons

**Affiliations:** Vitalant Research Institute, San Francisco, California, USA (I. Steffen, K. Lu, L.K. Yamamoto, G. Simmons);; University of California, San Francisco (I. Steffen, K. Lu, G. Simmons);; University of California, Los Angeles, California, USA (N.A. Hoff, A.W. Rimoin);; University of Kinshasa, Kinshasa, Democratic Republic of the Congo (P. Mulembakani, E.O. Wemakoy);; Institut National de Recherche Biomedicale, Kinshasa (J.-J. Muyembe-Tamfum);; Institute of Human Virology, Abuja, Nigeria (N. Ndembi);; Abbott Diagnostics, Abbott Park, Illinois, USA (C.A. Brennan, J. Hackett Jr.);; American Red Cross Scientific Support Office, Gaithersburg, Maryland, USA (S.L. Stramer);; Centers for Disease Control and Prevention, Atlanta, Georgia, USA (W.M. Switzer);; Institut National de la Santé et de la Recherche Médicale Unité 941, Paris, France (S. Saragosti);; Centre National de Transfusion Sanguine, Kinshasa (G.O. Mbensa);; Institut National de la Transfusion Sanguine, Paris (S. Laperche)

**Keywords:** Ebola virus, viruses, hemorrhagic fever virus, filovirus, hemorrhagic fever, serologic prevalence, neutralization assay, pseudotypes, luciferase immunoprecipitation system, ELISA, equatorial Africa, Republic of the Congo, Democratic Republic of the Congo, Cameroon, Uganda, Ghana

## Abstract

We conducted a serologic survey of 2,430 serum samples collected during 1997–2012 for various studies to determine the prevalence of the hemorrhagic fever virus Ebola virus (EBOV) in equatorial Africa. We screened serum samples for neutralizing antibodies by using a pseudotype microneutralization assay and a newly developed luciferase immunoprecipitation system assay. Specimens seroreactive for EBOV were confirmed by using an ELISA. Our results suggest a serologic prevalence of 2%–3.5% in the Republic of the Congo and the Democratic Republic of the Congo, which have reported outbreaks of infection with EBOV. In addition we detected a seroprevalence of 1.3% in southern Cameroon, which indicated a low risk for exposure in this region.

The 2014 outbreak of Ebola virus disease in West Africa has changed our understanding of viral hemorrhagic fever epidemiology. What was previously thought to be a sporadic, localized disease is now perceived as a more widespread threat to public health in heavily populated regions. The reemerging epidemic illustrated the need for epidemiologic investigations of the serologic prevalence and geographic range of hemorrhagic fever viruses, as well as development of novel serologic assays for their detection and surveillance.

Three species in the genus *Ebolavirus* and 1 species in the genus *Marburgvirus* within the family *Filoviridae* cause hemorrhagic fever in humans and have triggered several outbreaks with high case-fatality rates (*1*). Ebola viruses (EBOVs) have been found to asymptomatically infect different bat species and are known to cause fatal infections in great apes and other wildlife in the Congo Basin (*2*,*3*). Consequently, it has been proposed that bush-meat hunting, butchering, and consumption, as well as mining and caving, are potential risk factors associated with EBOV infection (*4*,*5*). However, epidemiologic links are not always well established (*6*), and the risk for exposure to hemorrhagic fever viruses for the general population in Central and West Africa remains unclear. Data are limited in that previous serologic surveys for filoviruses date back several decades (*7*–*9*) or have focused on specific populations and locations with high exposure to potential risk factors (*6*,*10*).

We conducted a widespread serologic survey for EBOV-specific antibodies in 5 countries in central Africa, including known filovirus-endemic regions (the Democratic Republic of the Congo [DRC], the Republic of the Congo, and Uganda), as well as areas without previously reported filovirus infections (Ghana and Cameroon). We tested 2,430 serum samples for specific antibodies to EBOV proteins to determine the seroprevalence of this virus in the respective populations. Serologic assays for detection of antibodies against EBOV glycoprotein (EBOV-GP), matrix protein (VP40), and nucleoprotein (NP) included novel microneutralization and luciferase immunoprecipitation system (LIPS) assays, as well as a commercially available ELISA.

## Materials and Methods

### Samples

**Figure 1:**
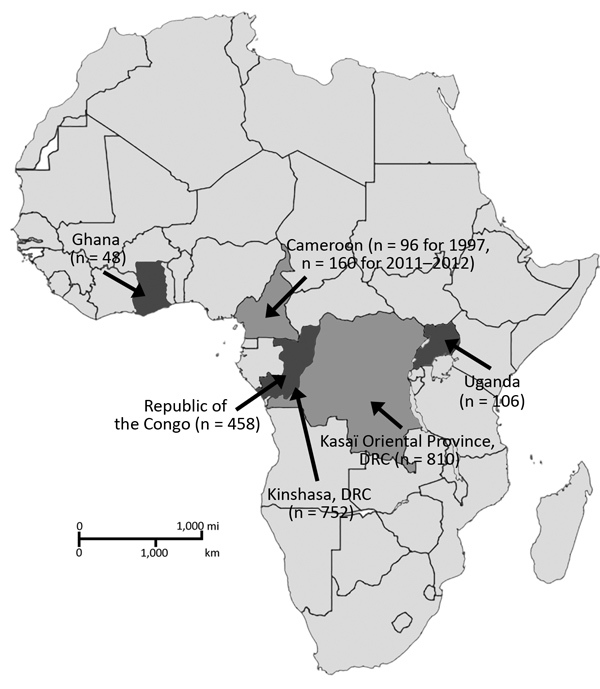
Numbers of serum samples collected from Ghana, Cameroon, Republic of the Congo, DRC, and Uganda in study of serologic prevalence of Ebola virus in equatorial Africa. DRC, Democratic Republic of the Congo.

The deidentified serum samples tested in this study were obtained from several different studies in 5 countries in Africa and were collected during different periods as part of unrelated surveillance projects using approved human subjects protocols. We obtained sampling locations, numbers, and collection dates (Table; Figure 1).

**Table:** Assay results for study of serologic prevalence of Ebola virus in equatorial Africa*

Country	City	Risk group	Collection period	No. samples	No. (%) EBOV neut+	No. (%) EBOV LIPS+	No. (%) EBOV ELISA+/no. tested	No. (%) EBOV confirmed	95% CI, %†
Uganda	Unknown	AIDS	1997	106	0	1 (0.9)	0/1	0	0–1 (0–0.9)
Cameroon	Unknown	AIDS	1997	96	0	4 (4.2)	0/4	0	0–4 (0–4.2)
Ghana	Unknown	AIDS	1997	48	0	0	0/0	0	0
Cameroon	All locations	Illness of unknown etiology	2011–2012	160	2 (1.3)	4 (2.5)	2/6 (33.3)	2 (1.3)	0–6 (0–3.8)
Cameroon	Djoum	Illness of unknown etiology	2011–2012	35	0	1 (2.9)	0/1	0	0–1 (0–2.9)
Cameroon	Ebolowa	Illness of unknown etiology	2011–2012	80	0	1 (1.3)	0/1	0	0–1 (0–1.3)
Cameroon	Sangmelima	lllness of unknown etiology	2011–2012	45	2 (4.4)	2 (4.4)	2/4 (50.0)	2 (4.4)	2–4 (4.4–8.9)
ROC	All locations	HIV surveillance	1999	458	4 (0.9)	9 (2.0)	9/13 (69.2)	9 (2.0)	0–13 (0–2.8)
ROC	Madingou	HIV surveillance	1999	149	1 (0.7)	1 (0.7)	1/2 (50.0)	1 (0.7)	0–2 (0–1.3)
ROC	Nkayi	HIV surveillance	1999	149	0	3 (2.0)	3/3 (100.0)	3 (2.0)	0–3 (0–2.0)
ROC	Owando	HIV surveillance	1999	160	3 (1.9)	5 (3.1)	5/8 (62.5)	5 (3.1)	0–8 (0–5.0)
DRC	Kinshasa	Blood donors	2011–2012	752	12 (1.6)	38 (5.1)	8/38 (21.1)	15 (2.0)	5–38 (0.7–5.1)
DRC	Kasaï Oriental	Monkeypox surveillance	2007	810	15 (1.9)	52 (6.4)	15/54 (27.8)	27 (3.3)	1–54 (0.1–6.7)
Total	NA	NA	1997–2012	2,430	33 (1.4)	108 (4.4)	34/116 (29.3)	53 (2.2)	6–116 (0.3–4.8)

*Confirmed seroprevalence rates are based on reactivity in >2 different assay formats. DRC, Democratic Republic of the Congo; EBOV, Ebola virus; LIPS, luciferase immunoprecipitation system; NA, not applicable; neut, neutralization assay; ROC, Republic of the Congo; +, positive.  †Based on number of samples reactive in all 3 assays to number of samples reactive in a single assay (values in parentheses).

### Cell Lines

Human embryonic kidney 293T, human rhabdosarcoma RD, and African green monkey COS-1 cells were grown in Dulbecco modified essential medium supplemented with 10% fetal bovine serum, l-glutamine, nonessential amino acids, and antimicrobial drugs (penicillin and streptomycin). Cells were incubated at 37°C in a humidified atmosphere containing 5% CO_2_.

### Plasmids

Expression plasmids were constructed by restriction-ligation cloning of viral glycoprotein sequences into multiple cloning site of the pCAGGS expression vector. We obtained the HIV pNL4–3 R^–^E^–^ plasmid containing the firefly luciferase gene (pNL-Luc) from the National Institutes of Health AIDS Reagent Program (https://www.aidsreagent.org). The corresponding plasmid encoding *Renilla* luciferase (pNL-Ren) was generated by swapping the firefly luciferase gene for *Renilla* luciferase (*11*). We also used the pRen2 vector for expression of *Renilla* luciferase fusion proteins (*12*).

### Pseudotype Preparation

We generated pseudotypes as described (*11*) by using 25 μg EBOV-GP expression plasmid or 30 μg Machupo virus glycoprotein (MACV-GP) expression plasmid and 10 μg pNL-Luc or pNL-Ren. Before use in neutralization assays, we titered pseudotypes for RD cells to normalize input relative light units.

### Pseudotype Microneutralization Assay

We diluted heat-inactivated human serum samples in culture medium, mixed with viral inoculum, and incubated at room temperature for 1 h. We used final serum dilutions of 1:50 and 1:500 in the high-throughput screen and dilutions ranging from 1:10 to 1:31,250 in titrations. Subsequently, 30,000 RD cells/well were added and plates incubated at 37°C for 48 h. The MACV-GP, which is from an unrelated arenavirus prevalent only in South America, was included as a negative control. All infections were performed in duplicate, and each plate contained identical controls, including uninfected cells, cells infected in absence of serum, and cells infected with virus incubated with negative control serum (US blood donor) or positive control serum from a survivor of Ebola virus disease. 

We lysed cells and measured luciferase activities in cell lysates by using the Dual-Glo Luciferase Assay System (Promega, https://www.promega.com). Infection rates in the presence of serum samples were expressed as percentage of infection in the presence of negative control serum.

Heat-inactivated human serum samples were diluted in culture medium, mixed with viral inoculum, and incubated at room temperature for 1 hour. Final serum dilutions of 1:50 and 1:500 were used in the high-throughput screen, and dilutions ranging from 1:10 to 1:31,250 were used in titrations. Subsequently, 30,000 RD cells/well were added and plates incubated at 37°C for 48 hours. The MACV-GP, which is from an unrelated arenavirus prevalent only in South America, was included as a negative control. All infections were performed in duplicates and each plate contained identical controls, including uninfected cells, cells infected in absence of serum, and cells infected with virus incubated with negative control serum (US blood donor) or positive control serum from a survivor of Ebola virus disease. Cells were lysed, and luciferase activities in cell lysates were measured by using the Dual-Glo Luciferase Assay System (Promega, https://www.promega.com). Infection rates in the presence of serum samples were expressed as percentage of infection in the presence of negative control serum.

### Luciferase Immunoprecipitation System

For expression of *Renilla* luciferase antigen fusion proteins, we cloned the C-terminal domain of EBOV VP40 (bp positons 583–981) into the pRen2 plasmid and transfected it into Cos-1 cells by using a 10-cm culture dish, 10 μg plasmid DNA, and the TransIT 2020 Transfection Reagent (Mirusbio, https://www.mirusbio.com). Cell lysates containing the fusion proteins were harvested at 48-h posttransfection. The LIPS assay was performed as described (*12*). We measured luciferase activities by using the *Renilla* Luciferase Assay System Substrate (Promega).

### ELISA

We obtained ELISA kits for detection of human IgG against Zaire EBOV NP from Alpha Diagnostics International (https://www.4adi.com). We performed assays according to the manufacturer’s instructions at 1:200 sample dilutions and read absorbance at 450 nm.

### Data Analysis and Assay Cutoff Determination

**Figure 2:**
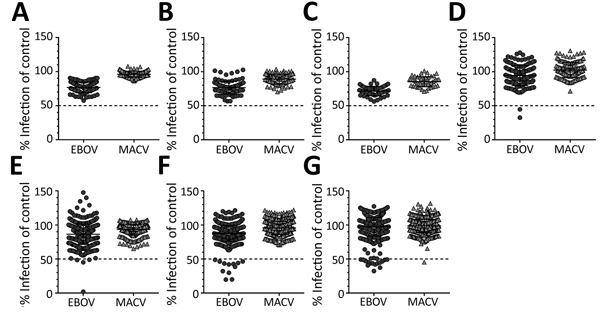
High-throughput screening data for neutralizing antibodies against EBOV and MACV glycoprotein pseudotypes in serum samples in study of serologic prevalence of Ebola virus in equatorial Africa. A) Uganda; B) Cameroon; C) Ghana; D) southern Cameroon; E) Republic of the Congo; F) Kinshasa, Democratic Republic of the Congo; G) Kasaï Oriental Province, Democratic Republic of the Congo. Serum samples were tested at a 1:50 dilution against the different pseudotypes. Samples that reduced pseudotype infectivity by >50% compared with a negative serum control were considered positive and confirmed by titration. Error bars indicate 95% CIs. EBOV, Ebola virus; MACV, Machupo virus.

We considered serum samples that reduced infection with EBOV pseudoviruses by >50% in the high-throughput screen compared with a negative serum control to be potential neutralizing samples and confirmed this assessment by titration over a 5-fold sample dilution series. MACV-GP pseudotypes served as internal control for nonspecific neutralization activity. Only 1 sample showed nonspecific neutralization of MACV pseudotypes at the 50% cutoff value, demonstrating the general specificity of the microneutralization assay (Figure 2).

We determined reactivity in the EBOV VP40 LIPS assay by calculating the cutoff for each experiment on the basis of the average + 3 SDs of 10 presumed negative samples from Kinshasa, DRC, included in each experiment to monitor plate-by-plate variations. We defined the cutoff value for the commercial EBOV-NP ELISA as 4.62 U/mL on the basis of the average + 3 SDs of the background signal in 47 presumed negative samples from Kinshasa. In addition, assay specifications require that the resultant 4 calibrator optical density values are plotted against concentration and that a linear fit be applied to the data. An R^2^ value >0.90 must be observed for the plate to pass this test. 

All assays included 3 serum samples from recently confirmed EBOV survivors as positive controls. Serum samples reactive in >2 separate assays were considered confirmed seropositive. We calculated prevalence rates on the basis of the number of confirmed seropositive specimens. We defined 95% CIs on the basis of the number of samples reactive in >1 assay (high) or reactive in all 3 assays (low).

## Results

**Figure 3:**
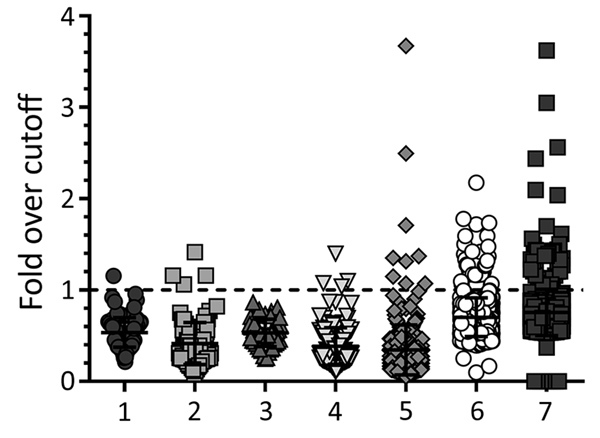
Antibody reactivity against Ebola virus matrix protein as measured by luciferase immunoprecipitation system assay for the different sample sets in study of serologic prevalence of Ebola virus in equatorial Africa. Data were normalized against individual cutoff values determined for each experiment. Samples yielding reactivity >1 were counted as positive specimens. Error bars indicate 95% CIs. 1, Uganda 2007; 2, Cameroon 2007; 3, Ghana 2007; 4, Cameroon 2011–2012; 5, Republic of the Congo; 6, Kinshasha, Democratic Republic of the Congo; 7, Kasaï Oriental Province, Democratic Republic of the Congo.

**Figure 4:**
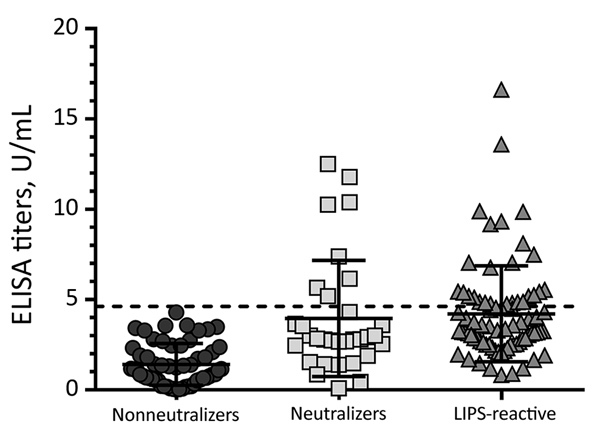
Summarized Ebola virus nucleoprotein ELISA data for confirmation of neutralizing and LIPS-reactive specimens across all sample sets in study of serologic prevalence of Ebola virus in equatorial Africa. For comparison, 57 random nonneutralizers were included. The ELISA cutoff value of 4.62 U/mL (dashed line) was determined on the basis of background reactivity for 47 serum samples from the local general population. Error bars indicate 95% CIs. LIPS, luciferase immunoprecipitation system.

We tested all samples for EBOV-specific antibodies against VP40 by using the LIPS assay (Figure 3) and for neutralizing antibodies by using a pseudotype neutralization assay (Figure 2). We then conformed seroreactivity in either assay by using an EBOV-NP–specific ELISA (Figure 4). No antibody-positive samples for EBOV were detected in the 1997 HIV surveillance samples from Ghana (n = 48) by any of the assays (Figure 2, panel C). The VP40 LIPS assay detected 1 (0.9%) of 160 reactive cases in Uganda and 4 (4.2%) of 96 reactive cases in Cameroon (Figure 3). However, none of these reactive samples were confirmed by neutralization assay or EBOV-NP ELISA (Figure 2, panels A, B; Figure 4). In contrast, samples collected in Cameroon during 2011–2012 contained 2 (1.3%) of 160 EBOV-neutralizing serum samples (Figure 2, panel D) and 4 (2.5%) of 160 VP40 antibody–positive samples (Figure 3). Both neutralizing serum samples were confirmed by EBOV-NP ELISA, but the 4 VP40-positive samples were nonreactive in other assays (Figure 4). Both neutralizing samples and 2 of the VP40-reactive serum samples were collected in Sangmelima, and the 2 other VP40-reactive samples were collected in Djoum and Ebolowa (Table).

Although analysis of the 1997 HIV surveillance samples from Ghana, Uganda, and Cameroon yielded inconclusive results, we determined an overall EBOV seroprevalence in Cameroon of 1.3% (95% CI 1.3%–3.8%) on the basis of the 2011–2012 samples (Table). Because both confirmed seropositive samples originated from Sangmelima, this finding resulted in a local seroprevalence rate of 4.4% (Table).

We tested 458 samples from 3 locations in the Republic of the Congo (Nkayi, n = 149; Madingou, n = 149; and Owando, n = 160) by using the VP40-LIPS and pseudotype neutralization assays. Four (0.9%) samples showed EBOV pseudotype neutralization activity: 1 sample from Madingou and 3 samples from Owando (Figure 2, panel E). Nine (2.0%) samples showed reactivity to VP40 in the LIPS assay: 5 from Owando, 3 from Nkayi, and 1 from Madingou (Figure 3). Although none of the 4 neutralizing samples could be confirmed, all 9 of the VP40-reactive specimens showed positive results in the EBOV-NP ELISA (Figure 4). The overall serologic prevalence for EBOV in the Republic of the Congo was 2.0% (95% CI 2.0%–2.8%) (Table). Local prevalence rates were 3.1% in Owando, 2.0% in Nkayi, and 0.7% in Madingou.

**Figure 5:**
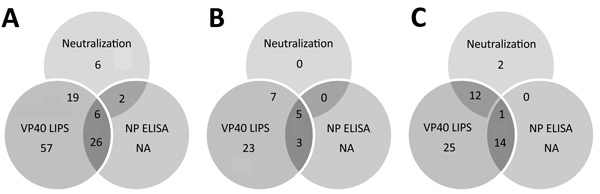
Overlap of different assay results for Ebola virus serology across all samples in study of serologic prevalence of Ebola virus in equatorial Africa. A) Total sample set; B) sample sets from Kinshasha, Democratic Republic of the Congo; C) sample set from Kasaï Oriental Province, Democratic Republic of the Congo. LIPS, luciferase immunoprecipitation system; NA, not applicable (ELISA was performed only for samples with positive results in other assays); NP, nucleoprotein; VP40, matrix protein.

Of 752 blood donor samples from Kinshasa, 12 (1.6%) showed neutralizing activity specific for EBOV-GP and were positive for VP40-specific antibodies by the LIPS assay (Figure 2, panel F; Figure 3). Five (41.7%) of 12 neutralizing specimens were confirmed by EBOV-NP ELISA, and they showed positive results in all 3 assays (Figure 4; Figure 5, panel B). Of the nonneutralizing samples, an additional 26 samples were reactive in the VP40 LIPS (total VP40 reactive = 5.1%), but only 3 samples were confirmed by EBOV-NP ELISA (Figure 3; Figure 5, panel B). The serologic prevalence for EBOV in this population was 2.0% (95% CI 1.1%–5.1%) (Table).

Similarly, of 810 samples from Kasaï Oriental Province, 15 (1.9%) samples neutralized EBOV-GP pseudotypes, but only 1 was confirmed by EBOV-NP ELISA (Figure 2, panel G; Figure 4). Thirteen of the 15 neutralizing samples (including the ELISA-positive sample) and 39 nonneutralizing samples were reactive with VP40 (total VP40-reactive = 6.4%) (Figure 3; Figure 5, panel C). Fourteen of the nonneutralizing VP40-reactive samples showed positive results in the EBOV-NP ELISA, which resulted in a triple-positive rate of 0.1% and a double-positive rate of 3.2% (Figure 4; Figure 5, panel C). The serologic EBOV prevalence in this population was 3.3% (95% CI 0.1%–6.4%) (Table).

We found a serologic prevalence for EBOV of 1.3% in Cameroon, a country without previously reported cases of EBOV infections, and prevalence rates ranging from 2.0% in the Republic of the Congo and Kinshasa (DRC) to 3.3% in Kasaï Oriental Province (DRC). We found no serologically reactive specimens in Ghana and Uganda in the samples available for this study (Table).

## Discussion

To better investigate the distribution of hemorrhagic fever viruses across Africa, we tested a large number of serum samples from 5 countries in East (Uganda), Central (Republic of the Congo and DRC), West (Ghana), and West Central (Cameroon) Africa, including samples collected during different decades. Our main concern after reviewing previously published serologic studies was an overestimation of seropositivity on the basis of data generated by assays with limited specificity. Therefore, we chose our testing strategy and defined our assay cutoff values in the most conservative way. The detection of neutralizing antibodies against viral glycoproteins is one of the most specific serologic tests for most viruses.

The microneutralization test enables simultaneous testing of serum samples against 2 distinct viral glycoproteins, thereby providing internal specificity controls. In our study, detection of EBOV antibodies by microneutralization might have led to an underestimation of seroprevalence because of a minor role of neutralizing antibodies in filovirus disease (*13*). However, one of our recent studies in EBOV disease survivors found neutralizing antibodies 40 years after exposure (*14*), suggesting a long-lived and robust response. Filovirus NPs resemble each other in amino acid sequence, organization, and structure among the different filoviral species and even show partial similarities to NPs of paramyxoviruses (*15*). Accordingly, studies have reported cross-reactive antibodies against NP with the highest frequency (*16*). In our study, we observed a high background signal in the EBOV NP ELISA when serum samples from areas with low risk for EBOV exposure were tested. This result led us to adjust the cutoff value for the NP ELISA to increase the specificity, which in turn reduced the sensitivity of this assay. Some studies suggest that antibodies against VP40 are more prevalent than antibodies against GP and NP in asymptomatic infections (*17*). However, there have also been reports of VP40 antibody responses in nonimmunized, presumably nonexposed humans, indicating some background immunity against EBOV in human populations (*18*). Our results suggest the presence of VP40 antibodies in persons who could not be confirmed to be specific for EBOV with any of the other antigens.

All samples were also screened for antibodies against Marburg virus (MARV) glycoprotein (MARV-GP); only 1 sample showed cross-reactivity against both filovirus glycoproteins. A small number of samples from all 5 countries included in this study were reactive against MARV-GP in a pseudotype neutralization assay and MARV-GP ELISA. However, these data could not be confirmed because of the lack of positive controls and available assays. Even so, this finding might suggest a wide geographic range of MARV or related viruses and their natural reservoir hosts. Serologic evidence for MARV infection of several bat species has been found in northern Republic of the Congo and Gabon, which have borders with Cameroon (*19*), and MARV RNA has recently been detected in bats in Sierra Leone (A. Liah et al., pers. comm.; [*20*]). Furthermore, a study that modeled the risk for zoonotic MARV transmission across sub-Saharan Africa found a substantial threat for MARV transmission in Cameroon and identified southern Cameroon as a beneficial target site for future surveillance efforts (*21*).

Serologic evidence for EBOV exposure was detected in the Republic of the Congo, the DRC, and the 2011–2012 samples from southern Cameroon. Although 10 outbreaks of Ebola virus disease have been reported in the DRC since its discovery in 1976 to its latest reappearance in 2018 (*22*), and a series of 4 separate outbreaks are known to have occurred in the Republic of the Congo during 2001–2005 (*23*,*24*), Ebola virus disease has not been diagnosed in Cameroon to date. However, a serologic survey conducted in 1983 in different areas of Cameroon suggested Ebola virus disease prevalence rates on the basis of indirect immunofluorescence (now known to be rather unspecific) ranging from 3.0% to 14.5%, depending on ethnicity and location (*7*). In comparison, we found a lower EBOV prevalence of 1.3% (95% CI 1.3%–3.8%) on the basis of reactivity with >2 virus antigens and different assay formats in a small set of samples from southern Cameroon. This finding illustrates the dependence of serologic data on sampling method, time, location, and assay specificities.

We also found EBOV prevalence rates of ≈2.0% for the Republic of the Congo and a blood donor cohort in Kinshasa, DRC. These results are consistent with those of a serologic survey of blood donors in the Republic of the Congo during 2011, which reported an EBOV prevalence rate of 2.5% (*5*). A serologic survey conducted during the 1995 EBOV outbreak in Kikwit, DRC, reported prevalence rates of 2.2% for workers in Kikwit and an average of 9.3% in surrounding villages, suggesting a higher risk for EBOV exposure in rural settings (*25*). Similarly, we identified a higher prevalence rate of 3.5% in villages in Kasaï Oriental Province in the central region of the country compared with a prevalence rate of 2.0% in the urban capital of Kinshasa. However, samples from the 2 cohorts in the DRC were collected as part of unrelated studies, under separate protocols, and at different time points, which limited the validity of direct comparison. Moreover, many persons living in Kinshasa have migrated to the capital from other parts of the country that have lower or higher risks for exposure to EBOV.

A study in the northeastern Watsa region of the DRC during 2002 reported one of the highest reported EBOV prevalence rates (18.7%) in the local Efé pygmy population, which included traditional primate hunters (*6*). These results, combined with our data, suggest that EBOV exposure risks might vary greatly in different locations and populations across Central Africa. Furthermore, the choice of serologic assay(s) and methods for setting assay cutoff values vary widely between different studies, which might contribute to the broad range of reported EBOV prevalence rates.

In conclusion, we used a conservative approach of multiple serologic assays with stringent assay cutoff values normalized by background assay reactivity for the general local population to determine that EBOV exposure outside recognized outbreaks is likely a rare event. Nevertheless, serologic evidence of past EBOV exposure was detected throughout different regions of central Africa. This finding could be explained by migration of persons from areas with known risk for exposure or thus far undetected presence of the virus in these regions. In addition, specific regions might experience increased levels of human exposure to EBOV or undiscovered related viruses because of ongoing environmental, societal, and behavioral changes.
